# Down-regulation of TM4SF is associated with the metastatic potential of gastric carcinoma TM4SF members in gastric carcinoma

**DOI:** 10.1186/1477-7819-9-43

**Published:** 2011-04-27

**Authors:** Zhouxun Chen, Suchen Gu, Bogusz Trojanowicz, Naxin Liu, Guanbao Zhu, Henning Dralle, Cuong Hoang-Vu

**Affiliations:** 1 Department of General Surgery, The first affiliated Hospital of Wenzhou medical School, Wenzhou 325000, Zhejiang, PR. China; 2 Department of General, Visceral and Vascular Surgery, Martin-Luther-University Halle-Wittenberg, Halle/Saale 06097, Germany

## Abstract

**Background:**

The aim of this study was to clarify the clinical significance of TM4SF members CD9, CD63 and CD82 in human gastric carcinoma.

**Methods:**

By employing RT-PCR and immunohistochemistry, we studied the expression of CD9, CD63 and CD82 in 49 paired tissue specimens of normal gastric mucosa and carcinoma. All tissues were obtained from patients who underwent curative surgery.

**Results:**

All normal gastric epithelium and gastric ulcer tissues strongly expressed transcripts and proteins of CD9, CD63 and CD82 as compared with corresponding controls. We found a significant correlation between CD63 mRNA level and different pM statuses (P = 0.036). Carcinomas in M0 stage revealed a stronger expression of CD63 than carcinomas in M1 stage. Expression of CD9 protein was found significantly stronger in pN0, pM0 than in advanced pN stages (P = 0.03), pM1 (P = 0.013), respectively. We found the relationship between CD63 expression, gender (p = 0.09) and nodal status (p = 0.028), respectively. Additionally, advanced and metastasized tumor tissues revealed significantly down-regulated CD82 protein expression (p = 0.033 and p = 0, respectively), which correlated with the tumor pTNM stage (p = 0.001).

**Conclusion:**

The reduction of CD9, CD63 and CD82 expression are indicators for the metastatic potential of gastric carcinoma cells. Unlike their expression in other tumor types, the constitutive expression of CD63 may indicate that this factor does play a direct role in human gastric carcinogenesis.

## Introduction

The TM4 superfamily (TM4SF) includes more than 20 core members and a number of additional proteins with sequence similarities. Nearly all mammalian cells contain one or more TM4SF proteins. The correct biological functions of the TM4 superfamily could not have been fully elucidated, but it has been reported that several TM4SF members, such as CD9, CD63, CD81, CD82 and CD151 might be involved in cell signaling. Furthermore, recent data suggest some TM4SF members might play roles in signal transduction pathways and to regulate cell activation, development, proliferation, and motility [1]. For instance, CD9, CD82 and CD63 have been reported to modulate the tumor progression or metastasis[2-4]. As type III integral membrane glycoproteins, CD9, CD82 and CD63 have two divergent extracellular loop domains, the larger of which contains several conserved amino acid motifs, highly conserved hydrophobic tetra-transmembrane domains and two short cytoplasmic domains at the NH2 and COOH termini[5,6].

CD9 gene is located on human chromosome 12p13.3 and encodes 227 amino acids. It was described originally as a 24-kDa surface protein of non-T acute lymphoblastic leukemia cells and developing B-lymphocytes[7]. CD9 is also expressed in plasma membrane of various cell types, including hematopoietic cells, endothelial cells, normal epithelial cells, and several tumor cell types[8-12]. Some clinical and experimental studies demonstrated that CD9 functions as a tumor metastatic suppressor in various cancers, including non-small-cell lung cancers, breast cancers, and colon cancers[13-15]. The CD82 gene is located on human chromosome 11p11.2 and encodes a 2.4 kb transcript which is translated into a N-glycosylated, transmembrane protein of 267 amino acids[3,16]. It attracted considerable attention as a tumor metastasis suppressor gene in prostatic cancer. Recent and retrospective studies have shown that decreased wild type CD82 expression could be a useful marker for metastatic and has invasive potential in some human tumor types, including pancreatic, breast, colorectal, bladder and oral cancers[17-23]. CD63 is isolated from human chromosome 12p12-q13 has been implicated in phagocytic and intracellular lysosome-phagosome fusion events. CD63 plays a role in the regulation of cell motility in melanoma cells and is involved in cell adhesion events[24], and strongly expressed on the cell surface in the early stage of malignant melanoma but weakly in the more advanced stages[25]. The data of our previous study demonstrated the expression of CD82 was correlated significantly with the metastatic status of human thyroid carcinoma. However, CD63 expression pattern did not correlate with any tumor staging[26].

The biological functions of these factors in human gastric carcinoma are still not clearly understood. In this retrospective study on staged human gastric carcinoma tissues, we investigated the expression of these three TM4SF members to determine whether they correlate with the invasiveness and metastatic ability of gastric carcinoma cells.

## Materials and methods

### Tissue specimens

No patient was required the perioperative neo/adjuvant chemotherapy in this study. From each patient, one representative primary tumor block, including tumor centre and invasion front as well as tumor-associated non-neoplastic mucosa, were examined by immuno-histochemistry.

Forty-nine patients were included in this study who with up to stage IV gastric carcinoma at the Department of General, Visceral and Vascular Surgery of Martin-Luther-University Halle-Wittenberg between 1994 and 2002. This study was approved by the local committee of medical ethics and all patients gave written consent. Tumor tissues were staged according to the Tumor-node-metastasis (TNM) staging classification (UICC-AJCC 1997). The clinical characteristics of the patients with gastric carcinoma are presented in Table 1.

**Table 1:** Relation between CD9, CD63 and CD82 expression and various prognostic factors

clinicopathological characteristics	No. of patients	CD9				CD63				CD82			
		**transcript **		**protein **		**transcript **		**protein **		**transcript **		**protein **	

**Gender**		***avarage***	***p-value***	***average***	***p-value***	***avarage***	***p-value***	***average***	***p-value***	***avarage***	***p-value***	*average*	***p-value***

Male	29	83.10	0.707	3.11	0.238	112.56	0.616	4.40	0.009	87.90	0.66	3.19	0.54

Female	20	82.31		4.06		110.12		3.00		80.47		2.64	

**Age**													

≤65	20	81.10	0.867	3.78	0.477	113.94	0.842	4.14	0.323	83.12	0.884	3.32	0.551

>65	29	83.97		3.27		109.83		3.54		86.00		2.74	

**Tumor stage**													

T1 and T2	13	85.14		3.75		114.23		3.73		91.38		4.00	

T3	11	89.98	0.79	3.43	0.215	107.64	0.462	3.17	0.81	78.34	0.866	2.39	0.033

T4	15	81.97		2.17		101.82		3.54		87.85		1.81	

**Nodal status**													

N0	5	74.61		5.60		105.13		4.25		67.82		4.40	

N1	13	79.73	0.556	2.91	0.03	106.47	0.774	4.23	0.028	77.85	0.23	2.88	0.094

N2	15	94.81		(N2 and N3)2,571		111.05		2.77		109.72		2.33	

N3	3	86.71				114.77		6.00		64.94		1.67	

**metastatic status**													

M0	11	90.68	0.403	4.64	0.013	121.84	0.036	3.90	0.137	90.40	0.77	4.35	0

M1	18	85.46		2.17		100.24		3.19		95.17		1.23	

**Differentiation**													

G1 and G2	5	81.93		4.20		118.89		5.25		83.67		3.75	

G3	24	85.77	0.82	3.05	0.624	108.33	0.432	3.31	0.105	87.57	0.691	2.23	0.304

G4	8	86.09		3.50		114.50		3.67		71.39		3.10	

**pTNM stage**													

I and II	12	79.10		4.08		109.53		3.91		73.67		4.06	

III	7	105.38	0.379	3.88	0.209	112.67	0.897	2.67	0.482	106.32	0.418	3.88	0.001

IV	19	69.66		2.50		96.80		3.68		46.35		0.95	

**Lauren's classification**													

intestinal type	12	69.30	0.105	3.42	0.538	109.93	0.719	4.50	0.06	80.17	0.773	3.80	0.535

diffuse type	22	91.26		3.03		112.63		3.41		84.80		3.41	

For employing Semi quantitative RT-PCR and immunohistochemistry, resected gastric tissues were immediately frozen in liquid nitrogen and maintained at -80°C. Frozen sections at 6 μm were cut by using Microm cryostat system (Microm International GmbH, Walldorf, Germany) on a cryostat and control sections were hematoxylin-eosin stained.

### Semi quantitative RT-PCR

To prevent crosscontamination of samples and carry-over contamination, laser-assisted microdissection was performed for subsequent isolation of genomic RNA (P.A.L.M.^® ^system, Bernried, Germany). Total RNA from fresh tissue samples, SW480 cell line (human colon carcinoma cell line) and FTC-133 (human follicular thyroid carcinoma cell line) was extracted by using the TRIZOL reagent (Invitrogen, Carlsbad, USA) according to the manufacturer's protocol. First-strand cDNA synthesis was performed with 1 μg of total RNA using a cDNA synthesis kit (Gibco, Munich, Germany) following the manufacturer's protocol at 42°C for 30 min followed by enzyme inactivation at 95°C for 5 min.

**Figure 1:**
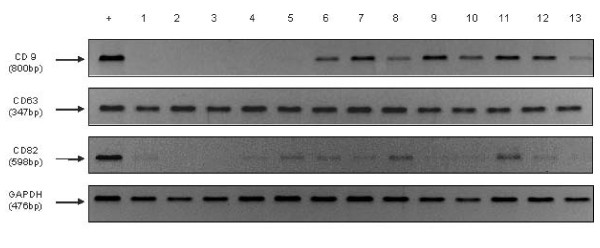
**1.5% Agarose gel electrophoresis of RT-PCR-amplified CD9, CD63, CD82 and GAPDH.** +: positive control; No.1-13: gastric carcinoma tissue samples

For PCR amplification, a 2 μl aliquot of the reaction mixture was used. The following PCR primer pairs were used to amplify a 800 bp amplicon of CD9 (sense 5'-TGCATCTGTATCCAGCGCCA-3'/antisense 5'-CTCAGGGATGTAAGCTGACT-3'; a 598 bp encoding CD82 (sense 5'- GCA GTC ACT ATG CTC ATG G-3'/antisense 5'-TGC TGT AGT CTT CGG AAT G-3') and a 347 bp amplicon for CD63 (sense 5'- CCC GAA AAA CAA CCA CAC TGC-3'/antisense 5'-GAT GAG GAG GCT GAG GAG ACC-3'), and a 467 bp amplicons for the housekeeping genes GAPDH (sense 5'-TGG TGA AGG TCG GTG TGA AC-3'/antisense 5'-TTC CCA TTC TCA GCC TTG AC-3'). All PCR reactions were performed with AmpliTaq (for CD9, CD82 and 18 S) and AmpliTaq-Gold (for CD63) (Amersham, USA). The PCR profile was as follows: 30 sec at 94°C, 45 sec at (CD9: 60°C; CD82:58°C; CD63:56°C, GAPDH:60°C) and 30 sec at 72°C. CD9, CD82, CD63 and GAPDH consisted of 30 sec at 94°C, 30 sec at 60°C, 45 sec at 72°C, and a final elongation step for 7 min at 72°C. 20 μl PCR products were run visualized in a 1.5% agarose gel (PeqLab), photographed with Kodak Image System 440 cf and electronically evaluated with "TL100" Total Lab software (Nonlinear Dynamics, UK). The expression of positive control was set as 100% (Figure 1), the expression levels of all investigated specimens were classified in comparison to the positive controls (for CD9 and CD63: SW480; and for CD82: FTC-133-CD82 overexpressing clone) grey scale. The densitometric values obtained for CD9, CD82 and CD63 bands in a given tumor tissue sample were divided by the corresponding value of GAPDH, and the ratio was referred to as the gene expression ratio for each gene. The evaluated value of a specimen between 0%- 20% was defined as "Negative"; 20%-50% "Decreased"; 50-75% "Moderate"; 75% and more "Positive".

### Immunohistochemistry

Immunohistochemistry was performed by using Dako Coverplates (Dako, Germany) on frozen tissue sections of 6 μm thickness. After 20 min fixation in a 1:4 mixture of 3% H2O2 in ice cold 90% Methanol, the slides were washed in phosphate-buffered saline (PBS) and pre-incubated for 10 min at room temperature with PBS - 1% bovine serum albumin (BSA), which was also used as a diluent for the antibodies. Successive sections were incubated overnight at 4°C with the CD9 (mouse monoclonal, MEM-61, abcam] at the dilutions of 1:200, the antibody against human CD82 (mouse monoclonal, clone 50F11, BD Pharmingen) at the dilutions of 1:300 and the antibody against human CD63 (mouse monoclonal, NKI/C3, Novocastra Laboratories Ltd) at the dilutions of 1:200, respectively. Negative control sections were only exposed to the secondary antibody and processed as described above. After 3 × 10 min washes in PBS, sections were incubated for 30 min with a 1:1000 dilution of biotinylated goat anti-mouse secondary antibody (Dako-anti-IgG-Kit) followed by incubation with an avidin-biotin-peroxidase complex. Specific immunostaining was visualized with a 15% diaminobenzidine (DAB) chromogenic solution (Dako, Aarhus, Denmark). Finally, sections were lightly counterstained with Mayer's hematoxylin. Tissue sections from a normal human tonsil (from patient who underwent tonsilectomy) were used as positive controls.

### Interpretation of immunostaining scoring

We employed the planimetric measurement features by using the "PALM RoboSoftware 3.2" (PALM MicroLaser Systems) software to determine the immunostaining intensity. This software allows the user to encircle areas for calculation (μm^2^). The sum of all immunopositive cell squares (μm^2^) was calculated and compared with the total section area. Subjective interpretation of immunohistochemistry was minimized by using a modification of the German immunoreactive score (IRS) method (Table 2). The immunohistochemical scoring was performed by two independent reviewers. A consensus opinion was used to score the rare cases for divergent opinions. We assigned an intensity score (0 to 3+) and a distribution score (estimated percentage of reactive cells) to describe staining of study cases. The criteria for scoring staining intensity were listed in table 2: To calculate the IRS, we assigned the following points for staining distribution: 1, 1-25% of cells; 2, 26-50%; 3, 51-75%; and 4, 76-100%. These points were then multiplied by the staining intensity score to give a range of potential IRSs from 0-12. Weak staining was defined as an IRS that ranged from 1 to 3, and moderate/strong staining was 4-12.

**Table 2:** Immunohistochemical scoring

A: Staining intensity	B: Precentage of positive Tumor cells	C: score
0 = no staining	0 = 0% positive cells	
	
1 = weak staining	1 =< 10% positive cells	
	
2 = moderate staining	2 = 10 - 50% positive cells	A × B = C
	
3 = strong staining	3 = 51 - 80% positive cells	
	
	4 => 80% positive cells	

### Statistical analysis

Sigmaplot 8.0 was applyed for all graphs calculations. Comparisons of the distributions of three TM4SF members expression for different groups were performed using the Wilcoxon-Mann-Whitney test (for two groups) or the Kruskal-Wallis test (for more than two groups). P-values of < 0.05 were considered to indicate statistical significance.

## Results

### CD9, CD82 and CD63 gene expression in gastric cancer tissues analyzed by RT-PCR

All normal gastric epithelium and gastric ulcer tissues strongly expressed transcripts of CD9, CD63 and CD82. Out of 49 gastric cancers tissues investigated, 17 carcinomas (34.7%) were evaluated as CD9 positive and 32 carcinomas (65.3%) as CD9 negative. Furthermore, 17 carcinoma tissues (34.7%) were evaluated as CD82 positive and 32 carcinomas (34.7%) as CD82 negative. Only 6 carcinomas (12.2%) were evaluated as CD63 negative, but 43 carcinomas (87.8%) were CD63 positive (Figure 1).

### CD9, CD82 and CD63 protein expression analyzed by immunohistochemistry

**Figure 2:**
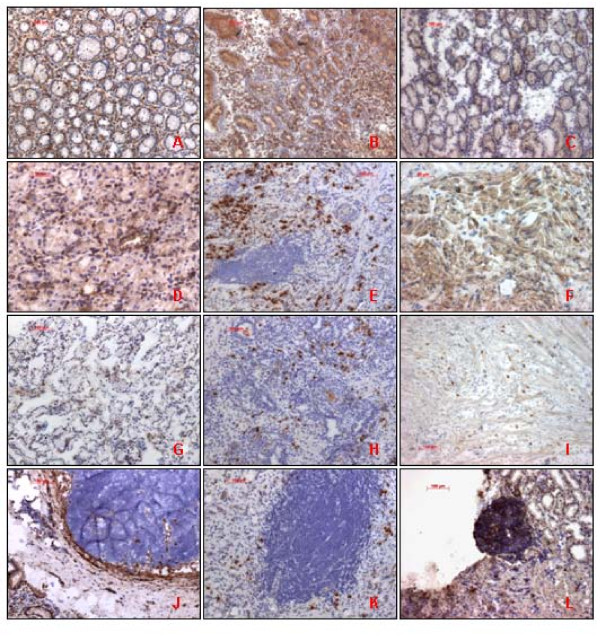
**CD9, CD63 and CD82 immunohistochemical staining patterns.** A,B,C: CD9, CD63 and CD82 expression in normal gastric mucosa; D, E, F: CD9, CD63 and CD82 expression in Gastric tumor tissue (non-metastasized); G,H,I: CD9, CD63 and CD82 expression in Gastric tumour tissue (metastasized); J,K,L:CD9, CD63 and CD82 expression in Lymph tissue (submucosa layer).

All normal gastric epithelium and gastric ulcer tissues were strongly expressed immunostaning of CD9, CD63 and CD82. Out of 49 gastric cancer tissues were studied by employing immunohistochemistry, 18 cases (36.7%) were classified as CD9 positive. In these cases, immunostaining of CD9 was intense and uniform on the cell-surface membrane (Figure 2). 31 cases (63.3%) revealed decreased CD9 expression, and the immunostaining in most of these tumors was heterogeneous. The immunohistochemical results were agreed with those of RT-PCR and 98.0% of the specimens coincided directly.

Further investigations demonstrated 21 CD82 positive cases (42.9%) and 27 CD82 negative cases (57.1%) (Figure 2). These results correlated with those of RT-PCR and 91.8% of the specimens coincided directly.

We identified 30 cases (61.2%) positive for CD63 and 19 CD63 negative cases (38.8%) (Figure 2). These results correlated with those of RT-PCR. However, only 73.5% of the specimens coincided directly.

### Relationship between CD9, CD82 and CD63 gene expression and various prognostic factors

The relationship between CD9, CD63 and CD82 gene expression and various prognostic factors are shown in table 1. Analysis of CD9, revealed no statistically significant correlations between gene expression and age, gender, tumor status, differentiation, pTNM stage and Lauren classification. Contrary, CD9 protein level was associated with lymph node status (p = 0.03) as well as with metastatic status (p = 0.013); Compared with 7(63.6%) of N1 stage patients and 11(68.8%) of N2-3 stage patients, no N0 stage patients showed negative gene expression. Furthermore, only 4(36.3%) of M0 stage patients had negative gene expression compared with 13(72.2%) of M1 stage patients.

The relationship between CD63 and various prognostic factors were associated with gender (p = 0.09) and nodal status (p = 0.028). Six male patients (20.7%) showed negative gene expression, and 13 female patients (65%) were CD63 negative. Furthermore, only one (25%) patients with N0 and 3 (27.3%) patients with N1 demonstrated negative gene expression compared with 9 (69.2%) of N2-3 stage patients.

In contrast, CD82 protein level was associated with tumor status (p = 0.033); metastatic status (p = 0) and pTNM stage (p = 0.001). 5 T1 patients(38.5%) were CD63 negative as compared with 6 T2 (54.5%) and 8 T3 (53.3%) patients. Analysis of metastatic status revealed that only 1 M0 patient (9%) and all M1 patients were CD63 negative. With respect to pTNM stage, only 2 stage I and II patients (22.2%) and no stage III patients were CD63 negative as compared with 10 stage IV (100%) patients.

## Discussion

In the present study, we investigated the expression of the three TM4SF members, CD9, CD63, and CD82. We demonstrated that CD9 protein levels were inversely associated with lymph node metastasis of gastric carcinoma. Furthermore, the reduction of CD9 protein was associated with distant metastasis of gastric cancer. Our results suggest that decreased levels of CD9 are strongly associated with an increased risk of recurrence, especially in patients with N0 nodal status and M0 metastatic status. These findings are consistent with previous reports demonstrating that reduced levels of CD9 correlated with poor prognosis of patients with breast and non-small cell lung cancers [13,27].

CD63 was originally described as a marker in the early stages of melanoma progression since it was highly expressed in radial growth-phase primary melanomas[28]. And it had been reported that it was strongly expressed on the cell surface in the early stage of malignant melanoma but weakens in the more advanced stages[25]. There is still no report showing that it is associated with other type of cancer. We demonstrated for the first time that mRNA levels of CD63 were associated with distant metastasis and CD63 protein correlated lymph node status. Taken together, these results suggested that decreased levels of CD63 were associated with a high pTNM staging and CD63 may served as a marker for metastatic potential of gastric cancer. Additionally, we found that CD63 proteins levels were lower in female that in male group. However, the reasons for that are still unclear and require further investigations.

CD82 has been identified as a metastasis suppressor gene[29]. Although the precise mechanisms for regulation of CD82 remain unclear, down-regulation rather than mutation is the most common mechanism in the progression of many cancers[14,17,22,30-33]. In our study, we demonstrated that the decreased levels of CD82 protein were strikingly associated with the tumor status and the distant metastasis. Furthermore, CD82 protein expression was inversely associated with the pTNM stages. These results were consistent with previous findings showing a direct correlation between reduction of CD82 gene expression and bad prognosis in patients with prostatic cancer and non-small cell lung cancer[3,34].

The mechanism of TM4SF mediated inhibition of cancer invasion and metastasis remains unclear. Cell adhesion and cell migration play important roles in a variety of physiological and pathological processes such as embryonic development, cancer metastasis, blood vessel formation and remodeling, and inflammation. Because of the specific structure of TM4SF[13], the extracellular domains can mediate specific protein-protein interactions with laterally associated proteins and unknown ligands, where they modulate integrin-dependent cell adhesion activities. The association of TM4SF members with various integrins is well documented. These members have little, if any, effect on the binding of integrins to their ligands or on integrin-mediated static cell adhesion to the extracellular matrix[35]. Instead, they regulate post-ligand binding events, including intergrin-mediated adhesion strengthening, a process whereby cells become increasingly resistant to detachment from immobilized integrin ligands[36].

The participation of TM4SF members in metastatic ability, morphological alternations and increased motility of tumor cells is often integrin-dependent. Previous reports demonstrated that the effects of CD63 on motility were similar to those reported for CD9 and both proteins were reported to associate with β1 and β3 integrins and to be identical with motility-related protein[36]. And previous work has also shown that the TM4SF members affect process such as cell proliferation, apoptosis and tumor metastasis. Various members of TM4SF associate with signaling enzyme, including protein phosphatases, conventional PKCs and type II phosphatidylinositol 4-kinase. CD82, as a suppressor of tumor cell metastasis, have the clearest mechanisms of the three members. the mechanisms of CD82 inhibits cell motility and invasiveness may because it can active the FAK-Src-p130Cas-CRKII pathway during cell migration and lead to DOCK180-dependent activation of Rac1 membrane ruffling and directional cell migration, with the p130Cas-CRKII complex functioning as a key "molecular switch"[37]. The association of CD82 with EWI2, another molecular that suppresses cell ruffling and migration[38], could also potentially contribute to the suppressor function of CD82. And CD82 attenuated signaling by the epithermal growth factor receptor (EGFR), and the related receptor ERBB2, by diminishing lig- and-induced dimerization and endocytosis[39]. This provides another avenue for CD82-mediated inhibition of cell migration and invasion.

For further understanding of the potential role of TM4SF in metastasis, we can go on to collect information of follow-up, and analyze the relationship of CD9, CD63 and CD82 with the Days after surgery. And in the future, we can also establish a gastric cancer cell model overexpressing CD9, CD63 or CD82, by using a plasmid vector. Then we can use the MTT test and Motility test to clarify relationship between wild type and the CD9, CD63 or CD82 overexpressing cells in cell proliferation and cell motility.

The classification of gastric cancers according to CD9, CD63 and CD82 expression might be useful in identifying patients for whom intensive adjuvant therapy is warranted. It is conceivable that testing tumors for TM4SF expression, in combination with other molecular and biochemical assays, may improve the prognostic evaluation of gastric cancer patients, and enhance the clinician's ability to prospectively identify patients who will have early disease recurrence and who require adjuvant chemotherapy. Many researches have shown that CD82 signalling may powerfully influence the development of metastasis. Although a significant amount of work is still required to uncover the mechanisms of action and regulation of CD82 in metastasis suppression, recent observations suggest that this metastasis suppressor gene and other members of this group of genes, like CD9 and CD63 will be of tremendous interest to the drug discovery industry for the development of therapeutics agents. It is conceivable that investigates the tumors for TM4SF expression, in combination with other molecules, may improve the prognostic evaluation of gastric cancer patients, and enhance the clinician's ability to prospectively identify patients who will develop early disease recurrence and who require adjuvant chemotherapy.

## Authors' contributions

The work presented here was carried out in collaboration between all authors. ZC and CH defined the research theme. ZC, CH, SG and BG designed methods and experiments, carried out the laboratory experiments, analyzed the data, interpreted the results and wrote the paper. HD, NL and GZ co-designed experiments, co-discussed analyses, interpretation, and presentation. All authors have contributed to, seen and approved the final manuscript.
